# Learning in friendship groups: developing students’ conceptual understanding through social interaction

**DOI:** 10.3389/fpsyg.2014.01031

**Published:** 2014-09-25

**Authors:** Carl Senior, Chris Howard

**Affiliations:** ^1^Department of Psychology (SW509b), School of Life and Health Sciences, Aston UniversityBirmingham, UK; ^2^Psychology Department, University of DerbyDerby, UK

**Keywords:** friendships, collaborative learning, student understanding, study groups

## Abstract

The role that student friendship groups play in learning was investigated here. Employing a critical realist design, two focus groups on undergraduates were conducted to explore their experience of studying. Data from the “case-by-case” analysis suggested student-to-student friendships produced social contexts which facilitated conceptual understanding through discussion, explanation, and application to “real life” contemporary issues. However, the students did not conceive this as a learning experience or suggest the function of their friendships involved learning. These data therefore challenge the perspective that student groups in higher education are formed and regulated for the primary function of learning. Given these findings, further research is needed to assess the role student friendships play in developing disciplinary conceptual understanding.

## INTRODUCTION

There is an extensive literature conducted from a range of theoretical perspectives and methodologies on the role of groups and student learning in higher education (see Haggis, 2009; Lundberg, 2014). The concept of the “group” is heavily contested within this literature with discrepancies in the formation, structure, size, duration, and function (Baron and Kerr, 2003; Forsyth, 2009). Despite this, within higher education (HE) practice, characterizing the “group” has tended to be more clear-cut. Groups of students are often constructed within the parameters of a particular educational program by tutors to address an explicitly defined learning objective (see Boud et al., 2001). From this perspective, student groups tend to be small scale (e.g., 2–5 members), function within the confines of the classroom and achieve tasks through cooperative or collaborative learning (Bruffee, 1993). Cooperative learning involves students dividing roles and responsibilities between group members, so learning becomes an independent process and outcome. On the other hand, collaborative learning involves students working together by developing shared meanings and knowledge to solve a task or problem (Dillenbourg et al., 1996, Dillenbourg, 1999). From this perspective, learning is conceptualized as a social process but also one that ultimately results in an individual outcome. That is, collaborative learning may facilitate individual conceptual understanding and higher-order thinking (Gillies, 2000).

The above perspectives on group learning both assume that groups are formed within the confines of formal learning environments (e.g., lecture theaters), involve students on the same degree program and have the explicit function of achieving a learning task. However, we have previously shown that student groups also tend to form spontaneously outside of the lecture room without the intervention of a tutor (Senior et al., 2012; see also Havnes, 2008); but, their function tends to remain centered on achieving an agreed and defined outcome by group members (e.g., the completion of a learning task). In this light, groups may disband once the task is completed by group members (Davies, 2009). The findings from the current study show that students use existing social networks such as friends as well as organized study groups as a mechanism for learning. Moreover, students may have used the social contexts in which they interacted with their friends outside of the classroom to further their understanding of disciplinary concepts in Psychology. However, the students did not conceive this to be a learning experience or suggest the function of their friendship groups involved learning. In this light, the current study suggests, in some contexts, students may not create, develop, and regulate groups for the function of learning as suggested in the literature (see Wenger, 1998; Borzillo and Probst, 2008; Orsmond et al., 2013) but use existing social groups as primae facie contexts in which to learn through social interaction. We refer to this as an “implicit community,” where tasks or events are achieved collaboratively but there is no awareness of the actual learning process or the subsequent outcome. This paper is divided into four sections: (1) theoretical accounts of student learning and groups; (2) the role of friendship groups and student learning; (3) discussion of the focus group methodology informed by critical realism which was employed to explore the role between groups and student learning; (4) the extent to which friendship groups regulated student understanding of disciplinary concepts (cognitive accounts of learning) or facilitated the development of disciplinary identities (social accounts of learning).

## STUDENT LEARNING AND GROUPS

Within the literature on student learning, cognitive approaches have tended to be the most influential with regard policy and practice (see Entwistle, 2001, 2009). From a cognitive perspective, learning is conceived in terms of information processing, achieved through the interplay of cognitive structures and processes (Marton and Pang, 2006). In this light, learning is construed as an individualistic outcome, best measured by the “depth” and “quality” of information processing. This underpins the distinction between “surface” and “deep” approaches to learning (Marton and Säljö, 1976), which has historically had a significant impact on the way in which the student experience has been analyzed, measured, and discussed (Richardson, 1990; Webb, 1997). Moreover, a surface approach involves superficial processing of information, which is categorized by memorization, whereas a deep approach involves a deeper level processing of information, which is characterized by conceptual “understanding” (Entwistle, 2001).

Over recent years in HE both undergraduate and postgraduate programs have tended to be designed to provide opportunity for students to work collaboratively and even in some cases across national boundaries (Dolmans et al., 2001; Keay et al., 2014; Rienties et al., in press). Here, students tend to be organized into small scale “groups,” which are designed to complete specific tasks that correspond to formalized learning objectives (Davies, 2009). This conceptualization of the “group” centers on Lewin’s (1948) notion of “interdependence,” where the success of individual group members is bound to the success of the group completing the task. The concept of the “group” in this context follows a stage driven approach, which is often employed in organizational settings (Reid and Hammersley, 2000). That is, the group forms for the purpose of completing a task, roles are assigned to group members, norms are established and the group disbands once its aim has been achieved (see Tuckman, 1965; Baron and Kerr, 2003). Within this framework, groups may employ principles from cooperative learning, where each member has a distinct role and largely works independently to achieve a task. Whereas, collaborative learning involves students working together, so roles may become interdependent or blurred (Bruffee, 1993). However, within these groups the emphasis is on the role of the course tutor who is central to the group development and hence its success as a potential learning device (Boud, 2001; Lancaster and Strand, 2001; Curseu and Pluut, 2013).

The evidence on the relationship between student groups and learning is encouraging from a pedagogic perspective. Barton et al. (2005) found students working in groups were more likely to score higher on an “openness to experience” scale that is significantly associated with a deep approach to learning (Zhang, 2003) compared to students who studied alone. Additional support was revealed by our previous work where it was found that students who completed a coursework task in a group significantly achieved a higher grade than students who completed a coursework task alone (Senior et al., 2012). The benefits of group work are such that it promotes “active” learning characterized by students engaging with a learning task and the development of wide portfolio of critical thinking skills (Gokhale, 1995). Group work may also increase students’ self-efficacy and motivation (Davies, 2009). Whilst the experience of working in groups may facilitate conceptual understanding, it additionally provides an opportunity to develop inter-personal skills which in turn may lead to an improvement in subsequent employability (Senior and Cubbidge, 2010; Senior et al., 2014) or as Mello (1993) argues, prepares students for the “real world” with the opportunity to develop social skills that are very likely to be required after graduation (see also Tymon, 2013).

Unfortunately student experiences working within such learning groups are not universally positive and some do report negative experiences. Those students who do not readily perceive the benefit of group work may not engage and subsequently interact with other group members (Walker, 2001). In turn, this may lead to negative outcomes such as “free riding” where some group members benefit from the accomplishments of others in the group but they do not contribute themselves (Salomon and Globerson, 1989). In the context of higher education, collectively a group may score high during an assessment designed to measure conceptual understanding but at an individual level “free riders” within that group may not understand the intended concept. This is problematic as this may produce a “sucker effect” where other group members respond to “free riding” by also becoming “free riders” themselves. Here, group work in HE may actually inhibit individual student conceptual development, which would require course tutors to carefully manage, design, and assess groups effectively.

Overall, from a cognitive perspective of learning, there is a literature that suggests working collaboratively may facilitate quality “individual” learning, which involves conceptual understanding. However, these groups tend to be organized by course tutors, have a distinct function on completing a specified learning task and disband once this is achieved. In light of this, it remains to be seen whether or not learning can occur in other forms of groups between students? One such social group that is ubiquitous throughout the HE sector are friendship groups.

## THE IMPORTANCE OF FRIENDSHIP GROUPS IN LEARNING

Early work has shown that friendship groups play an influential and significant role in the student life cycle (Spady, 1970). Students have been shown to use such social activity to develop cooperative learning strategies across a range of different classroom settings and while not all lead to equally effective strategies in learning most if not all such strategies are related to the development of a portfolio of transferable skills such as self esteem or the ability to work well with others (Slavin, 1988). This social skillset has been shown to play an effective mechanism in the facilitation of learning across a diverse student population (Hurtado et al., 2003). Interestingly there is an emerging body of evidence suggesting that the development of such learning strategies is also predicated by engaging within a friendship group on various social media platforms (Dabbagh and Kitsantas, 2012; Wang et al., 2012) which suggests that designers of distance learning provision should consider opportunities for students to engage with such activities as part of the online learning experience.

Within an HE setting and during the course of a campus based degree program students are likely to form and develop many diverse friendships with their peers on both their course and in the wider student community. According to Hartup and Stevens (1997, p. 355):

*“Friendship consists mainly of being attracted to someone who is attracted in return, with parity governing the social exchanges between the individuals involved. Friendships carry expectations that “best” friends will spend more time with one another than other persons, offering one another emotional support, including loyalty, trust, intimacy, and fun.*”

In light of the above quote, friendship groups may develop between students based on some form of mutual attraction, for example interests and even political values. Students may also form friendships with other students on their course for a variety of reasons including an interest in the discipline they are studying. Given the almost universal and pervasive nature of the friendship group within the student population it is incumbent on us to examine its utility, if any, as a potential learning device. The research literature with young children does suggest friendship groups positively impact on cognitive, emotional, and social development (Hartup, 1989) and aid psychological and affective adjustment to formal educational environments (Berndt, 1999). However, Antonio (2001) argues despite the growing literature on peer-to-peer interactions in HE, their still remains a need for research on the role of friendship groups within universities. The large-scale survey study conducted by the author above (*n* = 677) suggested that racially diverse student friendship groups were related to high levels of cultural awareness, racial understanding, and interracial interactions. Whilst this demonstrates the role between friendship groups and human relations, there still remains a lack of evidence on student learning. Nonetheless, Roberts (2009) conducted an ethnographic study of an undergraduate nursing program and found that friendship groups were used by students as a support mechanism where they could “ask anything” to develop their own understanding. This finding suggests that students who were categorized as friends were seen as a valuable source of knowledge, which was not subject to a hierarchical structure based on seniority or time served on the educational program. On the other hand, Antonio (2004) found students tended to use friends on their program of study as a “referral” point to judge their own academic competency. That is to say, friendship groups may be used by individual students as a mechanism to regulate their “academic self concept,” which refers to a student’s perceived academic ability (Rodriguez, 2009).

This process of social comparison may therefore impact on how students interact with their peers and their motivation to learn, which is associated with understanding of concepts (Entwistle and Waterston, 1988; Kahu, 2013; Mega et al., 2014). In this light, existing friendship groups between students do indeed impact on students learning. However, as noted above, while such groups are effective in ensuring that students do develop an “academic self concept” they are limited insofar as they suffer from the same constraints as the more formal tutor developed work groups, e.g., they tend to be defined for a specific purpose and will cease to function after their objectives have been met. However, while work has started to show that spontaneous friendship groups do indeed play an important role in the development of work based learning (Carr and Gidman, 2012) their efficacy within the HE sector and student learning has yet to be examined. The current research was therefore conducted as an exploratory study investigating the extent to which existing friendships between students may impact learning. These data would therefore provide an insight in whether learning in groups (such as friendships) can exist when the function of the group is not explicitly centered on completing a learning task and what this involves.

## METHODOLOGY

Two focus groups each lasting approximately one hour were conducted with seven first year students (for each focus group participants were split into a group of three or four, age range 18–20 years, five females and two males) enrolled on a Human Psychology degree at a UK higher education institution. Participants were randomly selected from a cohort of approximately 150 students. All procedures were approved by the local institutional ethics review board and all of the participants provided written informed consent prior to taking part in the focus groups. The sample size was deemed appropriate for the current study as it is consistent with the critical realist assumptions that underpin this study (see Parker, 1992) and with existing work in the field (e.g., Sims-Schouten et al., 2007; Easton, 2010).

The focus group schedule aimed to gather data on the students’ perceptions and experiences of learning. As the role of the researcher is one of a “moderator” or “facilitator” (Kidd and Parshall, 2000), there were three broad topics that were raised for group discussion: (1) What does a typical day at university involve? (2) What do you normally do outside of lectures and seminars? (3) What does learning mean to you and how do you know when you have learned something? In line with the principles of qualitative methods in psychological research it was important to use probing questions rather than specific leading questions on the role of friendship groups, as these may have shaped the responses of the participants in a socially desirable manner (see Willig, 2013). By using probing topics, this allowed the participants to draw upon their own lived experiences and discuss what was important and relevant to them (see Banister et al., 2011). Nonetheless, to ensure the focus groups addressed the role between student social interactions and learning, there were a series of prompts (i.e., What do you talk about with other students from university? Do you meet other students outside of university? What do you do together?) to direct the discussion.

Each focus group was conducted by one of the authors who had not taught the students or had any contact with the students prior to data collection, therefore minimizing social desirability artifacts. Data collection commenced during the final semester of the academic year, providing students with the opportunity to discuss the range of teaching methods, assessment, feedback, learning environments, and strategies experienced during the course of their studies.

The focus group data were transcribed by a research assistant and analyzed by the authors from a critical realist perspective. The key principles of critical realism are the existence of a real world which is multi-layered (ontology) produced by underlying causal mechanisms (epistemology; Bhaskar, 1975, 1979; Pawson, 1989). That is to say, mechanisms produce phenomena, which can then be experienced. Given this, as underlying mechanisms are unobservable due to the multi-stratified nature of reality, they can be inferred by exploring the similarities and differences in how people construct and add meaning to their experience of phenomena (Downward and Mearman, 2007). In this case, it is the experience of learning in groups during a first year Human Psychology undergraduate degree program. Despite this, the causal efficiency of a mechanism is regulated by a context (Lawson, 1997). Whilst mechanisms have the causal potential to produce phenomena; this may not be actualized within and across contexts. Within any educational context (e.g., lecture, seminar etc) there are likely to be a range of causal mechanisms that co-exist. This refers to an “open system” as the causal efficacy of a mechanism may be inhibited or actualized by other mechanisms within that context (Pawson, 1989; Sayer, 2000). In this light, research from a critical realist perspective becomes the process of inferring the causal mechanism(s) that may have produced the phenomenon under investigation and the contextual conditions in which these structures were realized. The emphasis is on the process of “inferring” mechanisms as these underlying causal structures are assumed to be unobservable; therefore they cannot be directly identified. As Sims-Schouten et al. (2007, p. 105) argue:

“*This means our attempts to identify and understand deep structures will remain just that – attempts. However, acknowledging that our knowledge of **reality** will always be limited is not the same as saying that there is no such thing as **reality**.” [our bold]*

However, evidence collected during an empirical investigation on participants’ experience of phenomena will draw upon the activity of mechanism(s), therefore aiding a researcher to make informed inferences and interpretations of causal mechanism(s). This process of inferring mechanisms is referred to as retroduction, which involves moving beyond description to underlying meaning (Pawson, 1989). In this light, inferred mechanism(s) borne from research data are more likely to be valid (i.e., correspond to actual mechanism∖s) than those developed from anecdotal or lay perspectives (see Benton and Craib, 2001; Carter and New, 2004). Whilst this research was exploratory, the aim was to examine the extent to which learning in friendship groups (proposed mechanism) may have facilitated student understanding (phenomena) during a first year Human Psychology degree (context). This was achieved by employing a “case by case” critical realist analytical approach. The qualitative data coding involved the process of observing variation within and between responses to develop themes. In this light, themes were used to identify similarities and differences in how the students constructed their experience of learning in friendship groups (both in and outside formal learning environments – lectures). From these data, within the context of this exploratory study we address the extent to which learning in friendship groups might be a mechanism for student learning from a psychological perspective. To ensure quality, the themes presented in the analysis were scrutinized by an independent expert in relation to richness and interpretation of data, depth of analysis and overall coherence (Parker, 1992; Elliott et al., 1999).

## ANALYSIS AND DISCUSSION

The first theme to emerge from the data was individual versus group learning. All the participants constructed learning as a cognitive outcome, which was best achieved through individualistic strategies to learn. Learning in the context of a group or with others was constructed as problematic because it prevented students from retaining facts. This is suggested in three extracts below:

“*I learn best on my own, I do the same as you (refers to a participant) I just summarize what was said in a lecture and read over it and over it till it sticks*.”

**Janet^**1**^**

“*I think ^1^All names reported throughout are pseudonyms.most of the time I study alone just because I prefer it like that, I think I would get less done in a group as discussion may stop me concentrating on learning the facts.*”

**Dave**

“*Personally I work best alone because I make a list of what I need to look at and tick it off one by one, make my notes and learn it, like I work best like that than trying to do it with other people, because then they can waste your time, like it’s not a waste obviously you are helping someone but you can give up a lot of your time to teach someone something you already know..*.”

**Zara**

These data suggest that learning was conceived as an individualistic cognitive outcome. Moreover, for Janet in the first extract, learning was constructed as the memorization of teaching materials, so successful learning involved accurate recall of information. Consequently, as evidenced in the extract by Dave, groups are conceived as problematic as social interaction may prevent students from retaining facts and hence learning. These data therefore suggest that psychology, as a discipline, is perceived by the students in the focus groups as dealing with concrete “facts” rather than concepts that are subject to debate. In the extract by Zara, learning is constructed as the transmission of information or facts. Overall, this suggests that learning was construed primarily as an individualistic process and outcome to which group work does not provide a facilitative context for this. This conceptualization of learning is largely problematic and challenges the perspective of some higher education practitioners who argue that learning is concerned with change and transformation rather than imparting “facts” or “truths” to passive students (see Prilleltensky and Nelson, 2002).

The second theme to emerge from the focus group data was collaborative learning through friendship groups. All the participants discussed the ways in which they interacted with other students on their course who they categorized as friends. These interactions suggested that learning was a social process as friendships provided contexts for participants to regulate how they learned, what they learned and to judge their success as a learner. The three extracts below illustrate the range of interactions participants had with their friends on the course.

*“...with my friends that are on my psychology course, I might have a discussion about...umm... whether we understand the stuff given from the lecture, we can then go through it together and have a discussion about it. Like I didn’t really get that lecture, my friend will go yeah I didn’t get it either. We have discussions and arguments about what has been said in lectures.*”

**Louise**

“*With my friends I talk about...um... lectures and then what we didn’t understand and then we’d like each read up a section and then try and explain it to one another and like we did that with one of our lectures and revising and stuff. I think that helps then because you know hearing from your friend is easier than hearing from someone you don’t know.*”

**Colin**

“*Within the exam period I talk with my friends quite a lot actually, like how much revision have you done? Or I might say oh I’ve done some today, yesterday and vice versa especially in exam period time then you talk more about exams. Coursework is exactly the same, well with me like, the coursework date is coming up soon, so I will say what you done and vice versa...um... you kind of go through it with each other and check*.”

**Sarah**

The above extract by Sarah suggests that interactions with friends were utilized as “reference” points during assessments to regulate learning. In this context, learning was positioned as a quantifiable measure, which could be used to judge how successful a student was by the amount of time they engaged with a task (e.g., revision and coursework). This extract suggests that interactions between friends provide a benchmark or measure to regulate how much time students spend on a learning task. In the extracts by Louise and Colin there is the suggestion that learning is centered on understanding concepts where social interactions between friends are utilized as contexts to facilitate this process. For both these participants, when they had problems understanding concepts from lecture material, their friendships became a resource to help develop their understanding. As evidenced in the extract by Colin, this strategy involved interaction and discussion between friends as they were seen as non-judgmental. Nonetheless, the form of interaction discussed in this extract between friends supports the earlier theme on individual vs. group learning. That is, learning through social interaction involved the transmission of information as a purely individualistic process and outcome. Interestingly, none of the participants explicitly discussed (nor when prompted by the interviewer) the interactions with their friends on their course (like those identified above) as learning experiences. Despite this, as suggested in these data, friendships may be an important aspect for learning during the first year of a degree program. In the extract below, Janet discusses how friendships between herself and other students on her course were developed.

“*All my friends on the course live at home like me but If I have just met someone doing psychology and they are telling me something, I don’t think I would listen but now I have been at unit for a long time and...umm... trust develops, so you become friends and then you can see how you can help each other and err, like helping with references. I wasn’t good with referencing then my friend helped me and like he wasn’t good at spelling and my other friend wasn’t good at setting out paragraphs so were just helping each other out.*”

**Janet**

The extract above suggests friendships were formed based on some commonality between students, in this case where they lived during term time. Despite this, for Janet, trust was critical to developing her friendships with other students and was achieved through regular interactions. Likewise, without trust Janet felt unable to accept the perspectives of other students. Friends therefore provide a support network to facilitate academic development at an individual level. Whilst only one participant discussed the formation of friendships with other students on their course, the extract does suggest that these groups are not developed for the purpose of learning but they provide a context for learning to occur once they are formed. Five of the participants also discussed how existing friendships in the wider student community (that is, outside of their course) were used to develop their conceptual understanding. This is suggested in the two extracts below:

*“...there are a lot of guys in my friendship group like you know... umm... when they are sat playing call of duty (video game) or something I am like this is going to make you, you know have more aggression due to the media and stuff just kind of like you know like chucking topics out there like or like we, I don’t know about you guys (refers to other participants), we did a lecture about nature versus nurture and media aggression and stuff like that it’s interesting to chat about with your friends really if it’s relevant to the modern day because friends not on my course will not know what I am on about but if you make it relevant to now then I can get a good discussion with my friends and see what they think and see if they are right*.”

**Suzy**

“*A couple of my friends were looking at a magazine and at the cover and images and things and I was saying like oh yeah about this and this, anorexia nervosa and this and they were like discussing with me. It was good to come up in conversation because I did this on my course and they were all listening and then talking about it. Also I have a friend who does optometry as well and she was talking about the vision in children and things and I joined in the conversation and she was like what are you doing this? I was like yep, yep I am learning this. It’s quite amusing really; it is good we got discussing it and I was getting a different view and starting to see what it all means*.”

**Zara**

The two extracts above both suggest interactions between friends facilitated conceptual understanding through the discussion and application of disciplinary concepts. In the first extract, Suzy applies theories of aggression (which were discussed in a lecture) to her friends’ “warfare” arcade game. This provides a learning experience, which enables Suzy through discussion with her friends to further her understanding of theoretical concepts by applying them to contemporary “real life” situations. The quality of this interaction therefore allowed her to add meaning and judge the validity of the theories involving aggression. In the second extract, Zara discusses two learning experiences with friends at University. The first involved discussion of eating disorders, whilst the second involved the visual perception of children. Interestingly, Zara makes reference to the fact that interacting with her friend who studied optometry provided a context in which she was given an insight into theories of vision from a different perspective (see e.g., Antonio, 2004). This experience provided scope for Zara to start to develop an interdisciplinary understanding of the concept of vision. However, integrating different disciplinary perspectives may not be valued across degree programs and may even have a negative relationship with student attainment. Nonetheless, these data are indicative of a deep approach to learning, characterized by an orientation to “understand” and extract “meaning” from a learning task (Entwistle, 2001). This finding therefore contrasts with the first theme, which suggested the participants tended to conceptualize learning in terms of memorization, which was often characterized as “retaining the facts.” These data therefore support the earlier argument that students did not view interactions with their friends as valid learning experiences, since learning was conceived in terms of retention and recall. This interpretation, however, remains tentative given the lack of data on how students explicitly discussed their conceptions of learning in relation to friendship groups. Despite this, the above extracts support the earlier argument that friendship groups were not formed based on the desire to achieve a learning objective but provided a context in which student understanding could be developed. However, these students did not necessarily demonstrate awareness that these interactions had a group function – conceptual development.

These data therefore provide evidence that existing friendships between students on a course and in the wider student community (“outside of the classroom”) were a resource in which the participants developed their understanding of theoretical concepts through discussion, explanation, and application to “real life” contexts. In the context of the current study, friendships may therefore have been an active mechanism facilitating student conceptual understanding. This process of collaborative learning is best understood as an “implicit community,” which refers to individuals achieving a task (in this context learning characterized as conceptual development) through social interaction but demonstrating no self-awareness. That is, people may feel that they are not part of a community or group but still achieve tasks by working collaboratively. This adds to the literature on groups (see Antonio, 2001, 2004; Baron and Kerr, 2003) by suggesting that groups may form and function through social interaction but membership may not be a conscious decision. Interestingly, all seven participants conceived learning as knowledge acquisition which involved retention and recall of course material. Furthermore, learning was perceived as “competitive” involving individualistic cognitive processes (retention strategies). Group work was therefore constructed as a problematic endeavor, as it prevented students from engaging in strategies to memorize facts (learn). This suggests the participants may not have seen the interactions they engaged with friends as valid learning experiences; we do, however, present this as a tentative interpretation of the findings given the lack of data directly addressing how students understood the relationship between friendship groups and learning.

The implications of the current research go beyond understanding the dynamics of student focused friendship groups as effective drivers of learning. The findings of the current study suggest that students may interact within such groups but not be immediately aware of the beneficial effect that such activity is having on their subsequent learning of various concepts. Such a finding would inform the current movement on the development of campus real estate that is designed to facilitate such social endeavors (Morrone and Workman, 2014). Initiatives such as the Primary Capital Program or the British Council for School Environments in the UK act as fora for innovation in the design of academic buildings for the tertiary education while initiatives such as the Learning Landscapes in Higher Education^1^ is an example of the emerging role that Architects and Educationalists can share together in the HE sector. When considered together with the findings of the current study it is clear that the design of any campus estate needs to incorporate the opportunities for students to meet in a social and non-directed capacity.

It is also interesting to speculate that such a learning mechanism may be used to design effective distance delivery. Specifically, with the regards to the development of Massive Open Online Courses (MOOCs) which often consists of many thousands of students taking part simultaneously. With regards to the design of such programs there is much debate as to the various means to support various learning styles (Grunewald et al., 2013) and program designers are now turning their attentions to various mechanisms that may engender and support a more community based style of learning (Gillani et al., 2014).

The data revealed in the current paper suggest friendships were formed with other students due to some form of mutual attraction, which is consistent with the exiting literature (Hartup and Stevens, 1997). It is important to note this mutual attraction may be centered on some aspect of learning (conceptions of, study strategies, etc.) but the friendship itself was not necessarily formed to specifically facilitate learning. Nonetheless, trust was seen as central to developing friendships and producing contexts where the social interactions between friends stimulated conceptual development at an individual student level. In this sense, collaborative learning was evident as students developed shared meanings and understandings through social interaction, which demonstrates learning at both an individual and social level (see Gillies, 2000). As the study was exploratory, involving two focus groups with a sample of seven first year psychology students, these findings are presented tentatively but they do raise a number of research questions that warrant further investigation adopting a longitudinal design: (1) how do friendships form and develop over the course of a degree program? (2) How do students understand and make sense of their friendships groups in higher education? (3) Are there differences between subject areas? (4) To what extent does the social interaction between friends relate to student conceptual development over the course of a degree? (5) To what extent does the interaction between friends relate to the development of disciplinary professional identities over the course of a degree?

## CONCLUSION

Within the context of this study, the focus group data suggested friendship groups may have been a causal mechanism for developing student conceptual understanding. Moreover, whilst students tended to conceive learning as an outcome involving memorization and perceived working in study groups as problematic (as it may prevent students from engaging in strategies to retain information), existing friendship groups provided a context to implicitly further students understanding of theoretical concepts. These friendships were not formed specifically to address a learning objective, which is often assumed from a psychological perspective but developed from some form of mutual attraction between students. The focus group data suggested that these friendship groups provided a setting in which trust was developed between students. Interactions between friends therefore created opportunities for students to explain disciplinary concepts, apply to “real life” situations and gain different perspectives, which may have facilitated conceptual understanding at an individual level. Given the study was exploratory, the findings were presented tentatively but they do suggest the importance of existing groups (not formed for the purpose or shared aim of learning) in developing student understanding. Future research therefore needs to address how friendships form, develop, and are understood by students over the course of a degree program along with the extent to which they produce a deeper conceptual understanding.

## Conflict of Interest Statement

The authors declare that the research was conducted in the absence of any commercial or financial relationships that could be construed as a potential conflict of interest.

## Footnotes

1 See http://www.rm.com/_RMVirtual/Media/Downloads/RM_PCP_White_Paper. pdf or http://www.bcse.uk.net/ or http://learninglandscapes.blogs.lincoln.ac.uk/files /2010/04/FinalReport.pdf for further information regarding these initiatives.
